# A Framework for Implementation of Dynamic Discrepancy Reduced-Order Modeling in Advanced Process Control

**DOI:** 10.1021/acs.iecr.5c02195

**Published:** 2025-12-23

**Authors:** San Dinh, Claudemi A. Nascimento, David S. Mebane, Fernando V. Lima

**Affiliations:** † Department of Chemical and Biomedical Engineering, 5631 West Virginia University, Morgantown, West Virginia 26506, United States; ‡ Department of Mechanical, Materials and Aerospace Engineering, 5631 West Virginia University, Morgantown, West Virginia 26506, United States

## Abstract

This paper introduces a novel framework for implementing dynamic discrepancy reduced-order modeling in advanced process control. This framework balances computational complexity and model accuracy by constructing a gray-box model that combines first-principles components with black-box functions to represent the critical process dynamics. Unlike conventional methods that correct plant-model mismatches based on output discrepancies, the proposed approach focuses on discrepancies in the rates of change within the reduced-order model. This method compensates for the reduced model’s loss of dynamic information, resulting in a more accurate model for process control. Three criteria are proposed for constructing dynamic discrepancy functions in gray-box models for model predictive control (MPC). Because differences in rates of change are not directly observable through the outputs, moving horizon estimation is used to guide data generation and collection for dynamic discrepancy functions. This technique allows flexible integration of dynamic discrepancies into the reduced-order model. Bayesian inference is employed to calibrate hyperparameters and apply the Occam’s razor principle to simplify the discrepancy functions. The approach is validated through a simulation involving a Fischer–Tropsch synthesis slurry bubble column reactor, demonstrating that the dynamic discrepancy approach can improve model and computational performance to be used in MPC applications.

## Graphical abstract

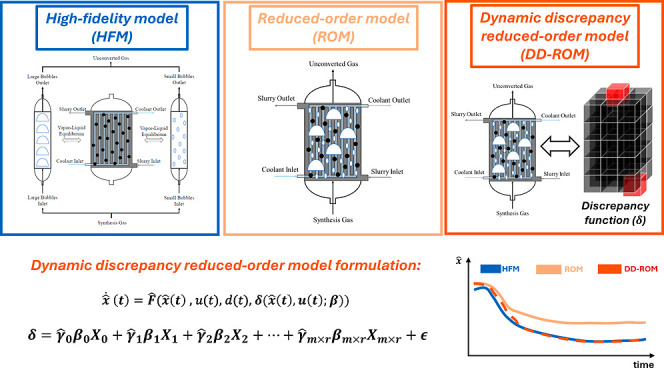

## 1 Introduction

In the recent developments in advanced process control, nonlinear model predictive control (NMPC) is an increasingly popular control strategy used in the chemical process industry and advanced manufacturing plants. Unlike linear MPC, which is designed for linear dynamic systems, NMPC handles nonlinearities in the system, including saturation effects, strongly nonlinear dynamics, large or frequent disturbances, convoluted input and output constraints, and operating conditions that vary widely and span a broad range of nonlinear process behavior.^1^ In NMPC, the nonlinear system model predicts the system future behavior and generates control actions that optimize a performance criterion over a specified prediction horizon. NMPC is often formulated as a dynamic optimization problem in which a mathematical model is used as an equality constraint set, and the objective is to find the optimal control actions. A well-known challenge in implementing NMPC online is the model’s computational performance, as the optimization problem must be solved within strict time constraints to enable real-time control, particularly in applications with fast sampling rates.

Several approaches have been proposed to address this challenge, including model reduction, algorithmic improvements,^2^ and hardware acceleration.^3^ While each approach has its own advantages and disadvantages, there is no universally applicable solution. The primary approach considered in this work is dynamic model reduction. Model reduction techniques for nonlinear dynamic systems have been extensively studied in the process control literature.^4–7^ One class of methods is projection-based techniques, where the system dynamics are reformulated in a reduced coordinate space. In this setting, less relevant dynamics may either be truncated or incorporated as algebraic constraints through residualization. Representative methods in this category include balanced model reduction^4,8–10^ and proper orthogonal decomposition (POD), also known as the Karhunen–Loève expansion.^11–13^ A second class comprises physics-based reduction techniques, which rely on process knowledge and physical insight to simplify models. These approaches are particularly useful for dynamic optimization, where computationally more efficient models are necessary.^14,15^ However, a major challenge when performing model reduction is the potential loss of accuracy, which may cause the optimum to deviate significantly from that of the original model.^16^

To overcome this challenge, the approach proposed in this work focuses on balancing computational complexity and model accuracy through a gray-box modeling framework. A more comprehensive high-fidelity model for control purposes may result in a small plant-model mismatch, which leads to improved closed-loop performance. However, such a detailed model increases the optimization problem complexity of the NMPC, and a longer time period is needed for the nonlinear programming solver to converge to an optimal solution.

The two most common mathematical model formulations for NMPC are the first-principles modeling approach and the black-box modeling approach. The first-principles modeling approach is based on a detailed understanding of the underlying physics and mechanisms of the system. This approach involves developing a mathematical model of the system based on fundamental principles, such as conservation laws, thermodynamics, and reaction kinetics. The obtained model is typically described by a set of differential equations, which can be solved numerically to predict the system behavior. In contrast, black-box modeling relies on empirical data and does not require a detailed understanding of the underlying physics or mechanisms. This approach involves developing a mathematical model of the system based on input–output data, using techniques such as system identification or machine learning. The resulting model is typically described by equations or empirical relationships, which can be used to predict the performance of the system.

The first-principles and black-box modeling approaches both have their own advantages and disadvantages. A first-principles model is generally more accurate, and it can be extrapolated to predict the system behavior under different conditions, whereas black-box models tend to have poor extrapolation capabilities. Alternatively, black-box models have superior computational efficiency, and their associated simplifications are systematic. These can follow either a parametric structure, defined by a fixed set of parameters, such as in linear regression and neural networks, or a nonparametric structure, where the number of parameters grows with the amount of available data, such as in Gaussian processes (GPs). In the model reduction, some key characteristics of the HFM are retained based on the process understanding, and the discrepancies between the HFM and the ROM are compensated by strategically placing black-box functions. This approach preserves the physics-derived properties of the ROM by embedding the discrepancy terms within the model parameters of the governing equations.

In the reported literature, discrepancy functions are often augmented to time-varying outputs.^17^ However, these output discrepancy functions are difficult to calibrate for a dynamic process because they require functional calibration inputs. To address this challenge, a dynamic discrepancy reduced-order model (DD-ROM) is developed in this work to reduce the model complexity for online control implementation. A DD-ROM structure was initially proposed to propagate uncertainty in a multiscale model.^18^ While formulating a DD-ROM is typically done on a case-by-case basis due to a diverse collection of model reduction methods, a set of heuristics for constructing a DD-ROM to model predictive control is provided in this work as a contribution.

The dynamic discrepancy functions correspond to black-box functions, and the identification of the functions is computed based on a Bayesian calibration framework. A special class of Gaussian process with Bayesian smoothing spline Analysis of Variance (BSS-ANOVA) covariance function is selected to fit the dynamic discrepancy because it can capture complex, nonlinear relationships among the system variables.^19^ Using Karhunen–Loève (K-L) expansion, the selected Gaussian process is decomposed into a sum of basis functions for model selection to avoid overfitting.

Another contribution of the proposed framework is a moving horizon estimation adaptation during data collection, which allows for the dynamic discrepancy function to take the form of Bayesian linear regression for more efficient calibration and model selection while maintaining the nonlinearities in the basis functions. Since the moving horizon estimation serves as a bijective mapping from the plant-model output mismatch to the dynamic discrepancy values, the formulations of the dynamic discrepancy functions are flexibly included in the reduced-order model.

Bayesian inference and modern control theory are the two fundamental aspects of the DD-ROM application to advanced control. Due to the common underlying mathematical framework, certain nomenclature is employed in both areas despite having distinct definitions. More specifically, the ambiguity of the terminology “inputs” and “outputs” is removed as follows. In the context of control systems, manipulated inputs are the variables or parameters that are directly modified by the controller to regulate the behavior of the system. These inputs can be physical quantities such as flow rates, pressures, or valve positions. In Bayesian calibration literature, the calibration inputs, which are sometimes referred to as control inputs, are the domains of the data-driven model. In a DD-ROM, the calibration inputs are the inputs of the dynamic discrepancy functions, which are a combination of the current state variables and the manipulated inputs. In a similar manner, the controlled outputs in this work are the variables that the controller is designed to regulate in order to achieve a desired objective, and the calibration outputs are the values of the discrepancy functions that aim to counterbalance the mismatch between the ROM and the HFM.

The proposed DD-ROM formulation is applied to obtain a simplified dynamic model of a Fischer–Tropsch synthesis reactor. The Fischer–Tropsch process is a chemical reaction that converts a mixture of carbon monoxide and hydrogen, called synthesis gas or syngas, into hydrocarbon waxes such as synthetic diesel, gasoline, and lubricants. Large-scale Fischer–Tropsch synthesis production is often carried out in a slurry bubble column reactor,^20^ which provides a large contact area between the catalyst pellets and the syngas, thereby increasing the conversion rate and overall efficiency of the process.

Overall, the main objective of this work is to propose a framework to construct dynamic discrepancy functions for reduced-order models employed in model predictive control. The gray-box modeling approach for advanced control implementation developed in this paper also provides an alternative to other offset-free closed-loop MPC strategies that address plant-model mismatch.^21–26^ To achieve this objective, the three major aims of the framework are augmenting the selected ROM with discrepancy functions, generating the data set that captures the differences between the ROM and the HFM, and calibrating the discrepancy functions. The remaining sections of the article are arranged as follows. The generalized system description and the mathematical formulation of the NMPC are provided in Section 2. A detailed description of the proposed approach and the criteria for the DD-ROM to be compatible with NMPC applications are discussed in Sections 3 and 4. Finally, a case study of the application of the framework to a Fischer–Tropsch synthesis process is demonstrated in Section 5, and the conclusions are outlined in Section 6.

## 2 Background

### 2.1 System Preliminaries

For high-fidelity modeling, a first-principles dynamic model is assumed with sufficient knowledge of the simulated system. This model is mathematically formulated as a system of nonlinear differential equations in the following form

1 $$ẋ\left(t\right)=F\left(x\left(t\right),u\left(t\right),d\left(t\right)\right),,x\left(0\right)=x_{0}$$

2 y(t)=H(x(t),u(t),d(t))

in which at any given time *t*, $x\left(t\right)\in R^{n_{x}}$ is the state vector, $ẋ\left(t\right)\in R^{n_{x}}$ is the vector of time derivatives of the states, $u\left(t\right)\in R^{n_{u}}$ is the manipulated/input vector, $d\left(t\right)\in R^{n_{d}}$ is the disturbance vector, and $y\left(t\right)\in R^{n_{y}}$ is the controlled/output vector. The dynamics of the high-fidelity model are described with the rate of change equations $F:R^{n_{x}+n_{u}+n_{d}}\to R^{n_{x}}$ , as presented in eq 1. The mapping $H:R^{n_{x}+n_{u}+n_{d}}\to R^{n_{y}}$ is a deterministic function that defines the output vector as a function of the state vector, the manipulated vector, and the disturbance vector, as shown in eq 2. It is important to note that, in this work, eq 1 represents a system of ordinary differential equations, with the time evolution of the dynamic model obtained by its integration.

In this work, the considered reduced-order dynamic model is a simplification of the high-fidelity model given above, and a more detailed discussion on model reduction methods is provided in the literature.^4–15^ A state-space representation of the reduced-order model considered is given by the following system of nonlinear differential equations

3 $$\dot{\hat{x}}\left(t\right)=\mathrm{F̂}\left(\mathrm{x̂}\left(t\right),u\left(t\right),d\left(t\right)\right),,\mathrm{x̂}\left(0\right)={\mathrm{x̂}}_{0}$$

4 ŷ(t)=Ĥ(x̂(t),u(t),d(t))

in which at any given time *t*, $\mathrm{x̂}\left(t\right)\in R^{n_{\mathrm{x̂}}}$ is the reduced-order state vector $\left(n_{\mathrm{x̂}}<n_{x}\right)$ , $\dot{\hat{x}}\left(t\right)\in R^{n_{\mathrm{x̂}}}$ is the vector of time derivatives of the reduced-order states given by the rate of change equations, F̂ . Since the reduced-order model is intended to replace the high-fidelity model in the NMPC formulation, it admits the same input vector and disturbance vector, *u*(*t*) and *d*(*t*), respectively from eqs 1 and 2. Additionally, the reduced-order model output vector, $ŷ\left(t\right)\in R^{n_{y}}$ , must contain the same variables as the output vector, *y*(*t*), of the high-fidelity model. Similarly to the high-fidelity model, the time evolution of the reduced-order model is solved by integrating eq 3, and the output, ŷ(t) , is obtained via mapping $Ĥ:R^{n_{\mathrm{x̂}}+n_{u}+n_{d}}\to R^{n_{y}}$ after a solution of x̂(t) is acquired, as shown in eq 4.

The dynamic systems defined in eqs 1–4 are assumed to be Lipschitz continuous. For a Lipschitz continuous system, the Picard–Lindelöf theorem guarantees the existence and uniqueness of the solution to every initial condition and a fixed path.^27^ This assumption is especially important for the dynamic discrepancy reduced-order model to be able to approximate the high-fidelity model in the following section. Furthermore, Lipschitz continuity is a reasonable assumption for physics-derived models as their state variables rarely experience extremely stiff transitions, i.e., trajectories that change rapidly and could destabilize numerical solvers. This assumption guarantees that such systems remain well-posed and facilitate the calibration problem. Additionally, the dynamic system of the HFM is assumed to be input-to-state practically stable. This assumption ensures that the effect of the disturbances on the state remains bounded and can therefore be recovered by the discrepancy function.

### 2.2 Nonlinear Model Predictive Control Formulation

The closed-loop system is simulated by consecutively solving the high-fidelity model within fixed-time intervals with zeroth-order hold on the control actions, and this task is carried out by a differential equation solver. In practice, NMPC is paired with a state estimator to determine the current state variables from the process measurements. Since state estimation is not the main focus of this work, full state information is assumed to be available to initialize the reduced-order model from the output measurements of the high-fidelity model at every time step. The schematic of the considered closed-loop system is illustrated in Figure 1.

**Figure 1:**
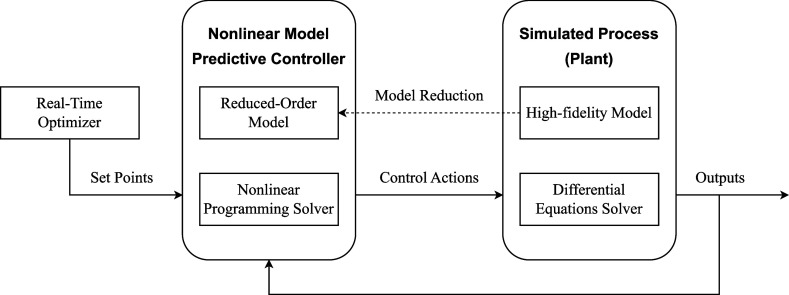
Closed-loop control with reduced-order modeling framework.

While the dynamics of the first-principles models are most accurately described in continuous time, process instrumentation and control equipment operate in the discrete-time domain, and thus the conversion between the two domains is necessary and is expressed as follows

5 $$t_{k}=\Delta t\times k,\forall k\in N$$

in which *t* is the continuous-time index, *t*_*k*_ is the discrete-time representation, and Δ*t* is the discretized time step. As a dynamic model can be rescaled appropriately by multiplying the rate of change eqs 1 and 3 with a global time scaling factor, without loss of generality, the discretized time step is assumed to be Δ*t* = 1 in the following NMPC formulation for ease of notations. This unifies the discretized time index at the *t*_*k*_^*th*^ time-step with the continuous-time representation at the moment *t*_*k*_.

For every *t*_*k*_, the considered NMPC is a set point tracking controller, and is formulated as the following constrained optimization problem

6 $$\min\sum_{k=1}^{N}\left\{{∥ŷ\left(k\right)-y^{sp}∥}_{Q}^{2}+{∥u\left(k\right)-u\left(k-1\right)∥}_{R}^{2}\right\}+{∥ŷ\left(N\right)-y^{sp}∥}_{P}^{2}$$

such that

7 x̂(k+1)=f̂(x̂(k),u(k))

8 ŷ(k)=ĥ(x̂(k),u(k))

9 $$\mathrm{x̂}\left(0\right)=L\left(x\left(t_{k}\right)\right)$$

10 c(x(k),u(k))≤0

in which ∥*s*∥_*H*_^2^ denotes the weighted sum of squares, *s* ^T^ *Hs*; *Q*, *R* and *P* in the objective function (eqs 6) represent the weight matrices of the predicted set point offsets, the calculated manipulated input variations, and the terminal cost, respectively, with all three matrices being positive definite to ensure that the optimal solution satisfies the second-order sufficient conditions. The vector of set points given by the real-time optimizer, *y* ^*sp*^, is obtained by solving a steady-state economic optimization subjected to the high-fidelity model in eqs 1 and 2. Zero-order hold is applied to the manipulated inputs, *u*(*k*), in between discretized time steps, and the discrete-time equivalent of the reduced-order model in eqs 3 and 4 is embedded in eqs 7 and 8. In the literature, there is a diverse collection of methods to explicitly discretize a system of nonlinear differential equations, such as the explicit Runge–Kutta methods. In the studies of dynamic optimization for NMPC applications, the discretization can be simultaneously solved with the optimal control actions by combining collocations or implicit Runge–Kutta methods into the constraints of the NMPC. Thus, the formulation of the discrepancy functions of the reduced-order model must be compatible with both discretization approaches. The equality constraint (eq 9) serves as the feedback component of a closed-loop system, in which the mapping *L* is the state estimation layer from the full state information, *x*, at the current time *t*_*k*_. The manipulated input, state, and output constraints are incorporated into the set of inequality constraints *c* in eq 10.

## 3 Dynamic Discrepancy Model Reduction Framework for Advanced Process Control

The dynamic discrepancy reduced-order model constructed in this section is a gray-box model. From an HFM, a reduced-order model is first established by using either a heuristic-based approach, a mathematical-based approach, or a hybrid between the two. In case the HFM is not available, empirical data could be used to obtain a purely black-box model that could be employed within an advanced process control technique. However, in this case, the proposed dynamic discrepancy-based method would not be applicable, as it requires to have at least a physics-based derived model portion. Black-box functions are employed to augment the reduced-order model and compensate for the truncated characteristics of the HFM, i.e., the simplification of the original high-fidelity model through the elimination of states or parameters. A model selection procedure is used to find the best formulation of the discrepancy functions according to Occam’s razor principle, which balances between model fitness and complexity. A schematic of the proposed framework is illustrated in Figure 2, for which the main steps are described below.

**Figure 2:**
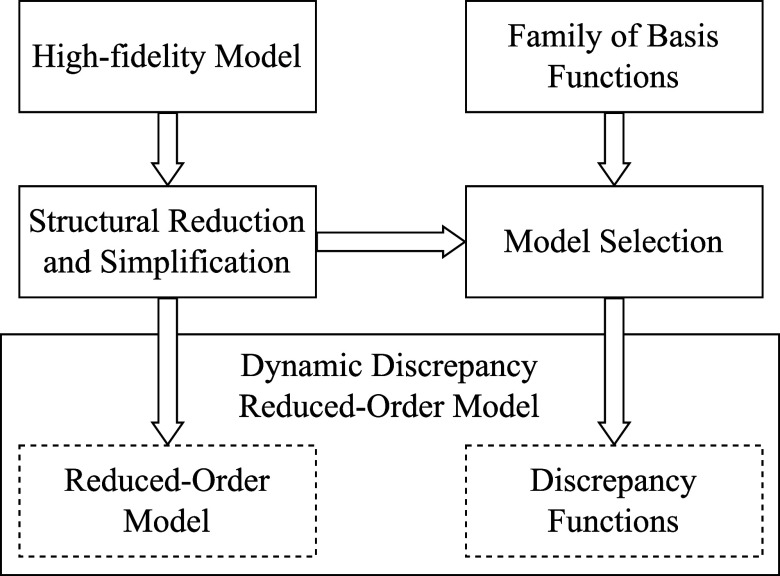
Dynamic discrepancy reduced-order modeling framework.

### 3.1 Dynamic Discrepancy

The formulation of a reduced-order model from a high-fidelity model inevitably removes or simplifies some components of the process model representation. Thus, discrepancy functions can be added to the reduced-order model to compensate for the differences from the high-fidelity model. For a dynamic process, a universal approach is appending the outputs from eq 4 with a black-box function as follows

11 $$\dot{\hat{x}}\left(t\right)=\mathrm{F̂}\left(\mathrm{x̂}\left(t\right),u\left(t\right),d\left(t\right)\right),,\mathrm{x̂}\left(0\right)={\mathrm{x̂}}_{0}$$

12 ŷ(t)=Ĥ(x̂(t),u(t),d(t))+δ(ŷ(t−1),ŷ(t−2),...,u(t),u(t−1),...;β)

in which the discrepancy function, δ, in eq 12 is a function of the past output measurements and the manipulated input sequence up to the current time step. Also, the vector of hyperparameters, β, is calibrated to match the transient behaviors of ŷ(t) and *y*(*t*).

The first challenge of the output discrepancy formulation in eq 12 arises from the mismatch in the time-domain inputs of δ and *ẋ*. By taking into account the dynamic behaviors of the system in the time interval preceding the current time step, the discrepancy function in eq 12 reflects more accurately the mismatch of the reduced-order model than a discrepancy function that only considers the outputs and manipulated inputs at the exact moment. However, if δ depends on every value of the manipulated input and the measured output sequences of the immediately preceding time intervals, then the discrepancy function is a functional instead of a function. In that case, the identification of δ is a search in a functional space, which is more computationally expensive than a parametric calibration of the hyperparameter β.

A solution for this challenge is considered here as a fixed and dense partition of the time interval preceding the current moment for the inputs of the discrepancy function. As a result, the rates of change in eq 11 are modeled in a continuous-time domain, and the inputs of the function δ in eq 12 are in a discrete-time domain. However, this approach leads to another difficulty when applying the reduced-order model to an NMPC. Since the embedded dynamic system in eqs 7–10 employs f̂ and ĥ, which are the discretizations of F̂ and Ĥ in eqs 11 and 12, a transformation of δ is needed before applying it to a closed-loop control system. Furthermore, dynamic optimization approaches to expedite the convergence of eqs 7–10 to the optimal solutions can be inhibited by the offline transformation of δ, and the NMPC is limited to the sequential discretization before optimizing solution methods.

An additional disadvantage of the output discrepancy formulation proposed in eq 12 is its convoluted calibration of the hyperparameters, β. Because the discrepancy function takes a form similar to a nonlinear autoregressive exogenous (NARX) model and the number of NARX’s inputs increases with the length of the considered input horizon, a large data set is required to reflect the transient dynamic behavior represented by the discrepancy terms accurately. Additionally, since the discretization time, Δ*t*, in eq 5 uniquely defines the calibration result of δ, tuning of the NMPC to a different update frequency of the manipulated inputs can require an updated data set and a new hyperparameter’s calibration.

The dynamic discrepancy reduced-order model is formulated here to address the above challenges associated with the output discrepancy dynamic model. In this formulation, the discrepancy function, δ, is incorporated into the governing equations of the reduced-order model. In particular, assuming that the dynamic systems in this work are Lipschitz continuous, and taking advantage of the Picard–Lindelöf theorem,^27^ the dynamic discrepancy reduced-order model matches the transient behaviors of the high-fidelity model by mimicking the rates of change of its state variables. Since the outputs are the projections of the state variables, the convergence of the reduced-order states leads to the convergence of the time-varying outputs to those of the high-fidelity model. Mathematically, a reduced-order model with dynamic discrepancy terms can thus be defined as shown in eqs 13 and 14.

13 $$\dot{\hat{x}}\left(t\right)=\mathrm{F̂}\left(\mathrm{x̂}\left(t\right),u\left(t\right),d\left(t\right),\delta \left(\mathrm{x̂}\left(t\right),u\left(t\right);\beta \right)\right),,\mathrm{x̂}\left(0\right)={\mathrm{x̂}}_{0}$$

14 ŷ(t)=Ĥ(x̂(t),u(t),d(t))

Because the dynamic discrepancy function, δ, in eq 13 only requires the current states and manipulated inputs instead of the past variables, the dimension of its domain is significantly lower than that of the output discrepancy function in eq 12. Consequently, if both the discrepancy functions are constructed from the same model architecture, the dynamic state discrepancy function is significantly more straightforward to obtain than the output discrepancy, and it thus has a lower potential to be overfitted when using the same data set. Additionally, the augmentation of the dynamic discrepancy function in the ROM is not limited to external additions, such as additive terms in the governing equations. It can also take the form of an internal enhancement, where the discrepancy function is incorporated into an existing system component or parameter.

Since there are many possible paths to obtaining the reduced-order model, the following step-by-step procedure, shown in Figure 3, was established to provide general guidance on building a DD-ROM. Step (i) involves analyzing key criteria before beginning the practical modeling of the DD-ROM. These criteria, outlined in Section 3.2, were heuristically defined and are introduced here to guide readers who are new to the topic, helping them avoid overlooking important aspects prior to the data collection and calibration of the DD-ROM. Step (ii) consists of the data collection procedure and is presented in Section 3.3. This step ensures that accurate and relevant data from the HFM and ROM are obtained and made available for the calibration step. The relevant data include the calibration inputs, comprising the reduced-order states (x̂(t)) and the manipulated input sequence (*u*(*t*)), as well as the calibration outputs, which correspond to the discrepancy values (δ­(*t*)) computed using a moving horizon parameter estimation procedure. Step (iii) focuses on calibrating the discrepancy functions (δ­(*s*)). While Section 3.4 provides the fundamental understanding of the BSS-ANOVA methodology used for training the discrepancy functions, further details on how to effectively calibrate the discrepancy functions and select the basis functions are presented in Section 4. This step is critical for ensuring that the DD-ROM can accurately mimic the HFM. Lastly, step (iv) consists of validating the resulting DD-ROM. The validation of the DD-ROM is a crucial step before its practical application. This is illustrated through the case study of the Fischer–Tropsch process, presented in Section 5.1.3.

**Figure 3:**
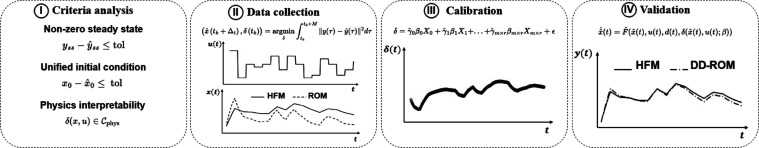
DD-ROM step-by-step building procedure schematic.

### 3.2 DD-ROM Construction Criteria

The construction of a dynamic discrepancy reduced-order model is considered here following some proposed heuristics. While a unified routine or algorithm to build a DD-ROM, especially to formulate δ, is difficult to generalize for all dynamic processes, the following fundamental criteria are proposed as heuristics for obtaining the DD-ROM of an NMPC:

1. Nonzero steady-state discrepancy: For all admissible manipulated inputs, the values of the dynamic discrepancies, δ(x̂(t),u(t);β) , must converge to unique nonzero steady states.
2. Unified initial conditions: The initial conditions of the DD-ROM and the HFM must be equivalent in nature, meaning that they must correspond and represent the same initial state.
3. Physics interpretability: Discrepancies should be added to physically defined components to safeguard the physics-derived properties of the ROM and to ensure that physics and thermodynamics laws are not violated.

The first criterion is necessary for the DD-ROM and the HFM to achieve similar steady-state outputs from the same inputs and can be imposed in the form of a constraint (e.g., $\left|y_{ss}-{ŷ}_{ss}\right|\leq \varepsilon$ ) evaluated prior to calibration of the discrepancy functions, requiring that the steady states of the HFM and ROM differ by at most a minimum amount given by a specified tolerance (ε). This criterion is critical for model predictive control applications. Because the considered NMPC is a regulatory controller, if the DD-ROM is unable to solve for the steady-state inputs associated with the given set point, the outputs of the closed-loop system will not converge to the desired targets. From the expressions in eqs 1, 3 and 13, it can be easily mistaken that the discrepancy terms, δ, equate to the differences between *F* and *F̂*. Because the HFM and the ROM are dynamically stable, one might consider applying corrections directly to the steady-state solutions of the ROM to achieve an exact match with the HFM through a linear combination of the rates of change. However, this approach would produce a dynamic discrepancy function that violates the first criterion and must be avoided. Instead, discrepancies should be applied to the dynamic states of the ROM, allowing the correct steady-state solutions to emerge naturally from the system dynamics. Additionally, the first criterion can be interpreted as a soft steady-state reset for the DD-ROM. Since the dynamic discrepancy functions alter the rates of change of the reduced-order model, without the uniqueness of their steady-state values, the plant-model mismatch becomes an integrated error with time. Different steady-state values allow the output mismatch to be locally bounded, even if the calibration of the discrepancy functions is imperfect.

The second criterion is recommended to reduce the offsets between the DD-ROM in the controller and the HFM plant, and can be imposed in the form of a constraint evaluated prior to calibration (e.g., $\left|x_{0}-{\mathrm{x̂}}_{0}\right|\leq \varepsilon$ ) requiring that the initial conditions of the HFM and ROM systems must minimally coincide according to a specified tolerance (ε). Since the dynamics of the systems are assumed to follow state-space structures that are modeled as initial value problems in differential equations, if the initial conditions of the DD-ROM and the HFM are not the same, the dynamic performances of the DD-ROM may be precise but not accurate in the best-case scenario. The dynamic discrepancy function assists the steering of the ROM dynamic states toward the solutions of the HFM, effectively correcting the ROM trajectory to match the HFM dynamics, but it does not directly incorporate past manipulated inputs or state variables. Thus, the DD-ROM is incapable of recorrecting itself once the initial states are misplaced, and identifying a proper initial condition is as crucial as calibrating the discrepancy functions. For a closed-loop system, the criterion on initial conditions is applied for the state estimation layer, *L*, in eq 9 using a similar rationale.

While the first two criteria focus on the numerical aspects that make DD-ROM compatible with the NMPC implementation, the third criterion seeks to preserve the advantage of the physical system by protecting the physics-derived properties of the ROM. For example, mass balances can be retained by incorporating the dynamic discrepancy into the kinetic parameters of reactions instead of directly adding the dynamic discrepancy to the mass accumulation terms. While augmenting an energy balance, it is advisible to enhance thermal conductivity or heat transfer coefficient with the dynamic discrepancy functions, rather than changing the heat of reaction or specific enthalpy. It is worth highlighting that the primary focus of this work is not on constructing the reduced-order model, but to compensate for the mismatch between a given reduced-order model and a high-fidelity model using the discrepancy functions. Given that parameters in the ROM may affect the system outputs by different orders of magnitude, sensitivity analysis can be employed to determine suitable locations for the discrepancy functions to be added in the ROM structure. This approach ensures the identifiability of the DD-ROM and its convergence toward reproducing the HFM profiles. Sensitivity analysis may be conducted using either local or stochastic/global methods.^6,28^ Overall, the dynamic discrepancy function is prioritized to represent the conversions over generations to ensure compliance with the physics conservation laws.

### 3.3 Data Collection

Data collection is critical in calibrating the dynamic discrepancy functions because it provides the necessary information, in this case, the reduced-order states (x̂(t)) , the manipulated input sequence (*u*(*t*)), and the dynamic discrepancy values (δ­(*t*)), for their calibration. The DD-ROM may not accurately represent the system without accurate and relevant data from the HFM, leading to inaccurate predictions or suboptimal solutions of the NMPC. The illustration of the data collection procedure is proposed in Figure 4, which is also suitable when an actual plant is used in place of the HFM.

**Figure 4:**
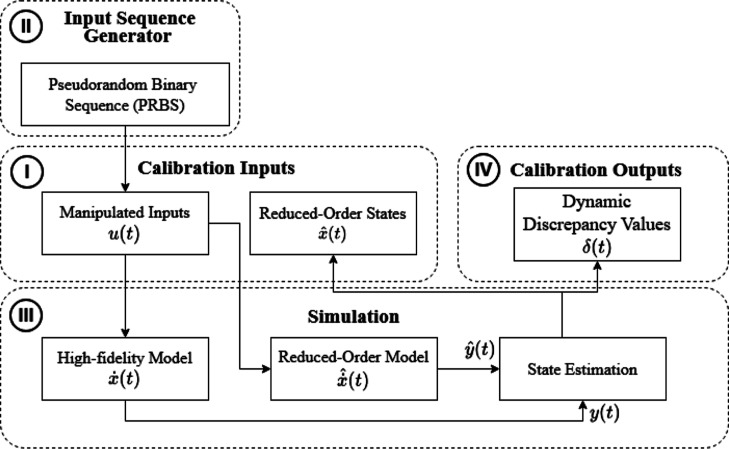
Calibration data collection method for dynamic discrepancy functions.

The proposed data collection procedure starts by defining which calibration inputs are going to be used, as shown in step (i). Because the discrepancy function, δ, has its domains containing *x̂*(*t*) and *u*(*t*), the calibration inputs are the states and the manipulated inputs of the ROM. Following step (i), step (ii) consists of generating a set of manipulated inputs that sufficiently excite the selected states in both the HFM and the ROM. To this end, a pseudorandom number generator, such as a pseudorandom binary sequence (PRBS), is used to generate manipulated inputs. System identification techniques can then be applied to design these inputs so that the system dynamics are adequately excited. Alternative approaches to generate the set of manipulated inputs include Gaussian or band-limited noise, multisine signals, or optimized input designs based on the Fisher information matrix.^29,30^ If the time to reach new steady states of the dynamic process is available, the holding period of the random manipulated input signal is chosen to be longer than this time. Because the dynamic discrepancy function cannot distinguish between a steady state and a transient state, choosing the period as such ensures the collected data characterize both scenarios. Additionally, the repetition of steady states in a calibration data set can act as a bias weight, meaning that it can skew the estimation of model parameters toward such particular operating points. This generated bias can be advantageous for NMPC applications as accurately reaching the set points lead to the desired outputs, which is more important than finding the optimal transient path.

In step (iii), given a specific manipulated input from the generated sequence, the HFM and ROM are simulated. Using the same input sequence for both models allows the calibration process to capture differences in system responses that may arise from mismatches in model structure or complexity. In step (iv), the calibration outputs, which correspond to the discrepancy values, δ­(*t*), in the DD-ROM model, are computed at each time step using a moving horizon parameter estimation procedure,^31,32^ as shown in eqs 15–18, that minimizes the RMSE between *y* and *ŷ*, employing an optimization algorithm. In this case, the dynamic discrepancy terms are treated as decision variables rather than functions, as in eq 13. This process continues iterating until the discrepancy values for the entire simulated time horizon are computed. Since the HFM model is independent of the calibration process, its output sequence resulting from the previously generated random signal is recorded as a reference for the moving horizon data generation for obtaining the DD-ROM, i.e., the moving horizon estimation updates the prior belief about the discrepancy values sequentially as the horizon moves forward. Thus, their optimal values in the estimation problem are used as calibration outputs to train the discrepancy functions (δ­(*s*)) in the calibration step. Further details of the calibration procedure are depicted in Section 4.
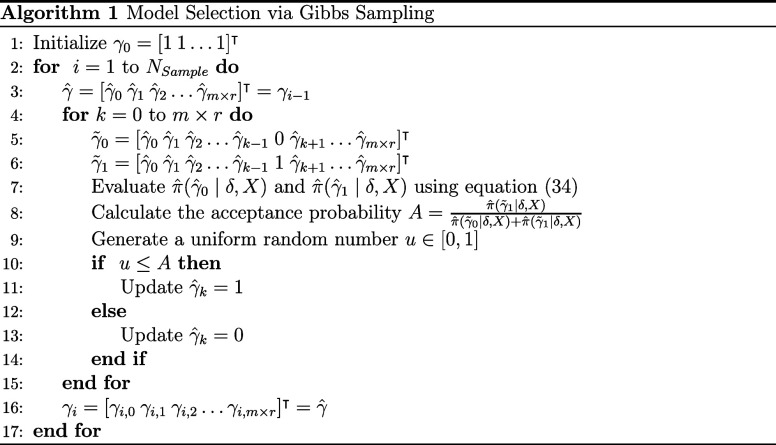


For each value of the manipulated input, the following dynamic programming problem is solved for each data point

15 $$\left(\mathrm{x̂}\left(t_{k}+\Delta_{t}\right),\delta \left(t_{k}\right)\right)=\underset{\delta }{\arg\;\min}\int_{t_{k}}^{t_{k}+M}{∥y\left(\tau \right)-ŷ\left(\tau \right)∥}^{2}d\tau$$

such that

16 $$\dot{\hat{x}}\left(\tau \right)=\mathrm{F̂}\left(\mathrm{x̂}\left(\tau \right),u\left(\tau \right),d\left(\tau \right),\delta \left(\tau \right)\right)$$

17 y(τ)=Ĥ(x̂(τ),u(τ),d(τ))

18 ĉ(x̂(τ),u(τ),δ(τ))≤0

In the parameter estimation problem formulated in eqs 15–18, the initial values of the DD-ROM are fixed as provided in eq 13, and included as a hard constraint in the first estimation problem associated with the first data point. Since the dynamic discrepancy function is added to the reduced-order model rates of change, the reduced state variables at the following discretized time step, $\mathrm{x̂}\left(t_{k}+\Delta_{t}\right)$ , depend on the value of the δ at the considered time *t*_*k*_. Thus, the solution of the state vector is used as an initial condition constraint in the following estimation problem, and they are also listed as calibration inputs.

The estimation horizon, *M*, in eq 15 is selected to be approximately the same as the time to reach a new steady state of the true dynamic process because any external perturbation to the state variables beyond this horizon has negligible effects on the states and the discrepancy terms. Furthermore, the selection of the horizon length is not affected by the size of the data set, thus maintaining the scale of the optimization problem in eqs 15–18 even for large data sets, which are required by high-dimensional calibration of input–output systems.

The inequality constraints in eq 18 include the physical constraints, such as nonnegative conditions for molar concentrations and choked flow limits for gases. Thus, formulating the calibration data collection as a nonlinear programming problem provides an additional benefit of filtering impractical data points. These constraints also prevent the dynamic process from operating in multiple steady-state regions. Because the dynamic discrepancy function is formulated as an explicit instead of an implicit function, each calibration input set only generates one set of calibration outputs. Output multiplicity behaviors, in which one set of calibration inputs can lead to multiple calibration outputs, lead to the precision of the calibration being significantly diminished.

The moving horizon parameter estimation in eqs 15–18 is defined in the continuous-time domain. However, this formulation does not invalidate the conversion of the DD-ROM to a discrete-time model while solving the nonlinear programming problem. Instead, it emphasizes the significance of transforming the dynamic discrepancy functions. If a dynamic optimization algorithm with embedded discretization, such as in the collocation method, is used to solve the estimation problem, its optimal solutions immediately contain the values of the calibration inputs. If a time discretization is performed explicitly for eqs 15–18 before a nonlinear optimizer solves the estimation problem, the optimal solutions would require an inverse transformation to achieve the correct dynamic discrepancy values in continuous time for the calibration inputs.

### 3.4 Basis Function Selection in BSS-ANOVA

In the following section, the dynamic discrepancy is assumed to be a Gaussian process (GP) with a Bayesian Smoothing Spline Analysis of Variance (BSS-ANOVA) covariance function. The BSS-ANOVA approach was originally introduced to address scalability issues often encountered in standard Gaussian processes by introducing a structured kernel.^33^ GPs are a powerful tool for modeling data sets and performing interpolation with uncertainty quantification, as they are composed of stochastic functions with broad nonparametric regression capabilities determined by their kernels.^34^ However, from a scalability perspective, training a GP requires O­(N^3^) operations due to the Cholesky decomposition of the full covariance matrix, which makes it computationally expensive for large data sets.^35^ Strategies like BSS-ANOVA help bridge this gap by handling the scalability challenge while preserving the predictive accuracy of GPs.

In the BSS-ANOVA approach, the kernel is first decomposed according to the ANOVA hierarchy, followed by a Karhunen–Loève expansion, resulting in the expression shown in eq 19, in which β_*j*_ and β_*j*,*k*_ are respectively the scalar coefficients of the main effect functions and the two-way interaction functions, and ϵ is the uncertainty introduced by the estimation of the parameters related to each basis function.

19 $$\delta \left(s\right)=\beta_{0}+\sum_{j=1}^{L_{\delta }}\beta_{j}\phi_{j}\left(s\right)+\sum_{j=1}^{L_{\delta }}\;\sum_{k=j+1}^{L_{\delta }}\beta_{j,k}\phi_{j}\left(s\right)\phi_{k}\left(s\right)+\cdot \cdot \cdot +\epsilon$$

The BSS-ANOVA framework has been shown to offer several advantages over the traditional Gaussian process approach.^36^ These advantages include the ability to handle categorical parameters in a flexible manner, as well as the capacity to model correlated outputs. Additionally, the BSS-ANOVA framework exhibits a linear computational complexity in the number of observations made by the simulator, thus further enhancing its computational efficiency.^37^ For these reasons, the discrepancy function in this work is going to be modeled as the expanded BSS-ANOVA shown in eq 19, where the calibration input, *s*, denotes the concatenation of the vector of reduced-order state variables, *x̂*, and the vector of manipulated inputs, *u*. Since the dynamic discrepancy values only depend on the calibration input at the same time instance, the data at each discretized time step is a pair of calibration inputs-outputs, and the time notation is dropped in the black-box model identification. While three-way or higher-order interactions can be included in eq 19, their effects are assumed negligible due to their infinitesimally small numerical values. In the Karhunen–Loève expansion, the domain of each basis function is fixed to the interval [0, 1] to avoid bias, and the calibration inputs are scaled to match this range with a bijective linear mapping. The covariance function of the BSS-ANOVA is additive in nature, in which the first two kernels are the first and second-order Bernoulli polynomials. The third kernel is a zero mean Gaussian process with the kernel: *K*(*s*, *t*) = – *B*_4_(|*s* – *t*|)/4!, so the number of nondegenerate eigenvalue-eigenfunctions is infinite. However, it is possible to estimate the eigenpairs by discretizing the domain of the covariance function and determining the associated eigenvalues and eigenvectors. The resulting approximations of the third and higher basis functions are represented in eq 20 and illustrated in Figure 5.

20 $$\delta \left(s\right)=\beta_{0}\phi_{0}\left(s\right)+\beta_{1}\phi_{1}\left(s\right)+\beta_{2}\phi_{2}\left(s\right)+\cdot \cdot \cdot +\epsilon$$

**Figure 5:**
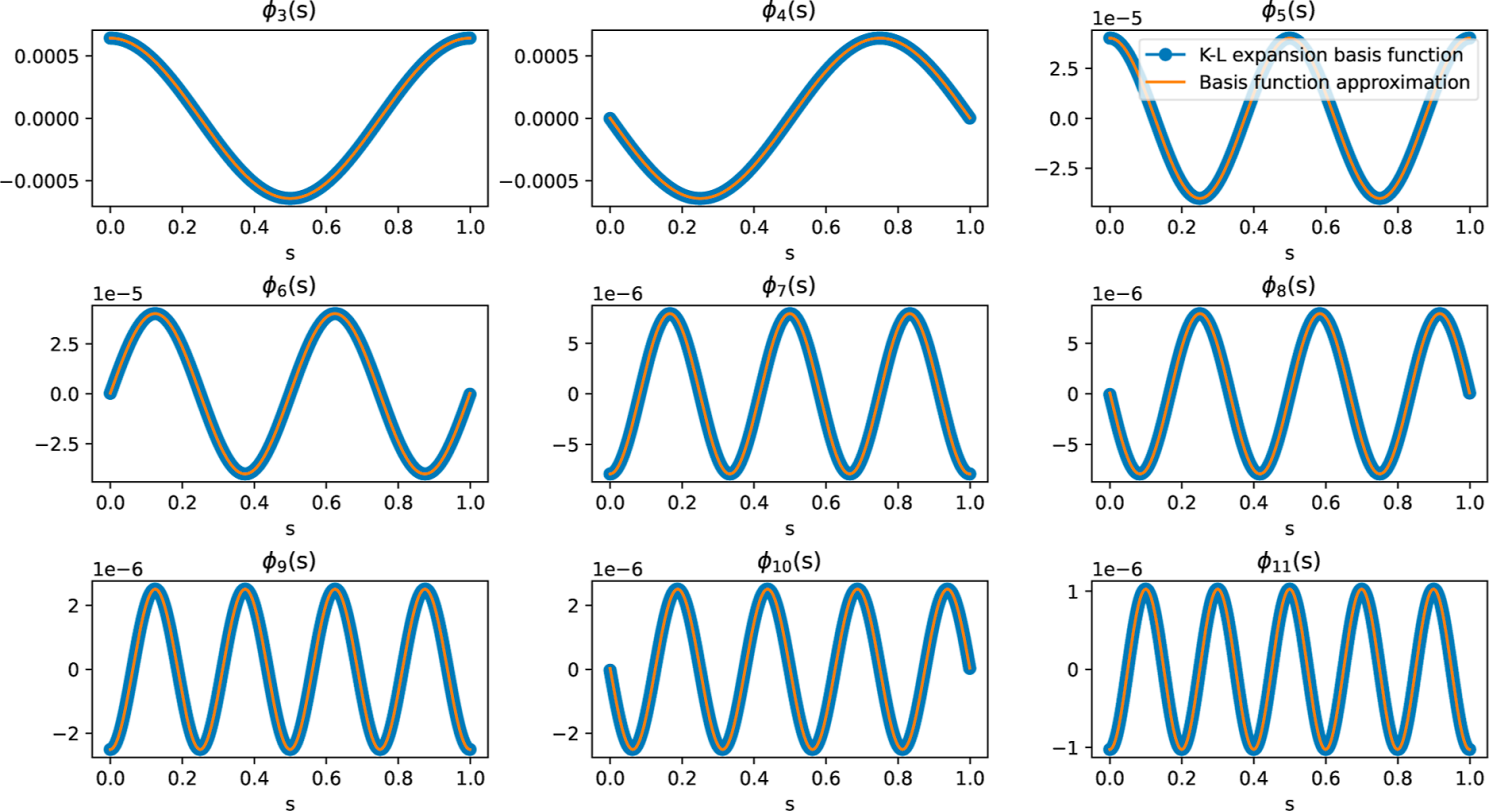
Approximations of basis functions for the decomposed BSS-ANOVA Gaussian process.

The discrepancy model calibration in the following section is a Bayesian inference technique. Although this method can flexibly find the posterior distributions of the hyperparameters without the proposed moving horizon estimation, it requires a very intricate likelihood function. In practice, a closed-form expression of the likelihood function of a nonlinear model is unlikely to exist, and it is commonly approximated by a Monte Carlo simulation,^38^ an Approximate Bayesian computation (ABC),^39^ or a kernel function.^19^ Monte Carlo simulations and ABC methods are computationally expensive, so they may not be suitable for large-scale systems. The kernel function typically requires an additional layer of Bayesian inference on its hyperparameters, which makes the calibration of the posterior of β more complex. However, the kernel is fundamentally a way of quantifying the differences between the data and the expected behaviors, such as the integrated square error in the objective (eq 15) of the proposed moving horizon estimation. Thus, the proposed approach is a hybrid that benefits from the robustness of numerical optimizations in the data collection while preserving the flexibility of Bayesian inference in identifying hyperparameters and model selection. Additionally, the estimation layer separates the vector of dynamic discrepancy values into independent calibration problems. The correlations between different dynamic discrepancy values and the DD-ROM are indirectly embedded in the optimality of the estimation problem. Therefore, instead of calibrating all discrepancy functions under the same posterior function, the calibration of each term can be done independently and in parallel with each other.

## 4 Calibration of the Dynamic Discrepancy Terms of the Reduced-Order Model

The main objective of this section is to outline the calibration method used for the dynamic discrepancy terms of the ROM. In Section 3.4, a limit on the number of basis functions to be considered in the calibration process was imposed, as shown in Figure 5. Given the nature of these terms, the task now can be understood as identifying the best black-box model based on a data set of input–output pairs. Although various methods have been suggested in academic literature, this study primarily employs the Bayesian model selection techniques outlined by Del Moral et al.^38^ The proposed calibration method relies on Bayesian principles, which allow for a probabilistic evaluation of different models to determine the most suitable one. By incorporating prior knowledge and updating it with new data, this approach provides a robust framework for model selection in the context of dynamic discrepancy terms.

### 4.1 Bayesian Model Selection

Bayesian calibration and least-squares estimation are two different approaches used to estimate model parameters from observed data. While neither approach is inherently better than the other, Bayesian calibration can offer certain advantages over least-squares estimation in certain situations. Bayesian calibration has the advantage of incorporating prior knowledge or beliefs about parameters into the estimation of parameters, thereby helping to reduce uncertainty. This is particularly useful when the available data is limited or noisy, as the prior information can provide additional constraints on the parameter values. By computing the posterior distributions of the parameters, Bayesian calibration can also establish a framework by which the uncertainty associated with parameter estimates can be quantified. As a result, it is possible to select a mode with a greater degree of confidence, as a range of parameter values and their associated probabilities can be provided. The Bayes’ Theorem is stated as follows

21 $$\pi \left(A|B\right)=\frac{\pi \left(B|A\right)\pi \left(A\right)}{\pi \left(B\right)}$$

in which π­(*A*|*B*) denotes the probability density function of a set of variables *A* given fixed values of a set of variables *B* and vice versa. The right-hand side of eq 21 is often referred to as the posterior density function. The likelihood function is the conditional density function, π­(*B*|*A*), and the prior is the probability density, π­(*A*). Since the posterior density function is required to be integrated into one, the denominator, π­(*B*), is the normalization constant of the numerator over the domain of the set *B*. In a Markov chain Monte Carlo method, which is later used to sample the probability of the discrepancy model over the given calibration data set, only the product of prior and the likelihood is required. Thus, an expression of the posterior density function that is not normalized is sufficient to calibrate a black-box model.

From the data generated using the procedure in the previous section, let *n* be the number of data points, *m* be the number of basis functions considered for each calibration input, and *r* be the number of calibration inputs. Consequently, the *i*th calibration inputs of the *j*th data point is denoted *s*_*i*,*j*_. The matrix of calibration inputs, *X*, is constructed as follows.

22 $$X=\left[\begin{array}{lllllllll} 1 & \phi_{1}\left(s_{1,1}\right) & \phi_{2}\left(s_{1,1}\right) & ... & \phi_{m}\left(s_{1,1}\right) & \phi_{1}\left(s_{2,1}\right) & \phi_{2}\left(s_{2,1}\right) & ... & \phi_{m}\left(s_{r,1}\right)\\ 1 & \phi_{1}\left(s_{1,2}\right) & \phi_{2}\left(s_{1,2}\right) & ... & \phi_{m}\left(s_{1,2}\right) & \phi_{1}\left(s_{2,2}\right) & \phi_{2}\left(s_{2,2}\right) & ... & \phi_{m}\left(s_{r,2}\right)\\ 1 & \phi_{1}\left(s_{1,3}\right) & \phi_{2}\left(s_{1,3}\right) & ... & \phi_{m}\left(s_{1,3}\right) & \phi_{1}\left(s_{2,3}\right) & \phi_{2}\left(s_{2,3}\right) & ... & \phi_{m}\left(s_{r,3}\right)\\ \vdots & \vdots & \vdots & ... & \vdots & \vdots & \vdots & ... & \vdots \\ 1 & \phi_{1}\left(s_{1,n}\right) & \phi_{2}\left(s_{1,n}\right) & ... & \phi_{L}\left(s_{1,n}\right) & \phi_{1}\left(s_{2,n}\right) & \phi_{2}\left(s_{2,n}\right) & ... & \phi_{m}\left(s_{r,n}\right) \end{array}\right]$$

in which the first column of ones is added to the calibration inputs to represent the bias of the chosen black-box model and the generated data. Since each basis function of each calibration input can either be added to or removed from the dynamic discrepancy function, the number of possible models for calibration is 2^*m* × *r*+1^. An effective method of enumerating every possible model is using a vector of indicators $\gamma ={\left[{\mathrm{\gamma ̂}}_{0}{\mathrm{\gamma ̂}}_{1}{\mathrm{\gamma ̂}}_{2}...{\mathrm{\gamma ̂}}_{m\times r}\right]}^{Τ}$ , in which each ${\mathrm{\gamma ̂}}_{i}\in \left\{0,1\right\}$ . Additionally, let the number of selected bases in a model γ be *n*_γ_, which is also the sum of all indicators in γ. The dynamic discrepancy function is reformulated as shown in eq 23.

23 $$\delta ={\mathrm{\gamma ̂}}_{0}\beta_{0}X_{0}+{\mathrm{\gamma ̂}}_{1}\beta_{1}X_{1}+{\mathrm{\gamma ̂}}_{2}\beta_{2}X_{2}+\cdot \cdot \cdot +{\mathrm{\gamma ̂}}_{m\times r}\beta_{m\times r}X_{m\times r}+\epsilon$$

If, and only if, all the components in ${\mathrm{\gamma ̂}}_{i}=1$ , then the basis function of the calibration input corresponding to the *i*th column of the matrix of calibration inputs, *X*, is included in the model associated with γ. For each uniquely defined model γ, the respective vector of hyperparameters, β_γ_, is a subdivision of β that only contains the *i*th components, β_*i*_, if ${\mathrm{\gamma ̂}}_{i}=1$ . Similarly, the truncated matrix of calibration inputs, *X*_γ_, is the matrix that only contains the *i*th column, *X*_*i*_, if ${\mathrm{\gamma ̂}}_{i}=1$ . The calibration error is assumed to be a zero-mean independent and identically distributed Gaussian random vector with variance ε^2^. Let *N*(μ, Σ) be the Gaussian distribution with a mean vector μ and a covariance positive definite matrix Σ, and let *I* denote an identity matrix with appropriate dimensions.

The objective in this subsection is to construct the overall posterior probability, π­(γ|*X*, δ), of any model γ given calibration data *X* and δ. From a process systems engineering perspective, the posterior probability of a model can be interpreted as a measure of the extent to which the dynamic discrepancy function fits the performance of the HFM instead of a statistical probability. Furthermore, the probability of a model is also closely related to the concept of model complexity, which measures the number of basis functions or degrees of freedom in the model. By balancing the complexity of the model with the fit to the data, the probability of a model provides a way to avoid overfitting and identifies accurate and computationally inexpensive models. This balance is achieved by constructing the probability of the model using a Bayesian framework.

In the following Bayesian model selection, noninformative priors are selected to avoid subjective bias and to place an equal chance for all possible models before the calibration data is incorporated. In particular, Zeller’s noninformative g-prior^40^ is selected to introduce a penalty term that encourages simpler models. The hyperparameters are represented by a Gaussian distribution with a mean of zero and independent and identically distributed variances that are proportional to the inverse of the sample size, multiplied by a constant called the g-prior. The g-prior is denoted as *c* in eq 24 and is set to a value between 0 and 1, with smaller values indicating less informative priors. The Jeffrey prior is used in eq 25 for the variances of moving horizon estimation errors since it remains unchanged when the model parameters are transformed.^41^

24 $$\beta_{\gamma }|\gamma ,\varepsilon^{2},X,c∼N\left(0_{\gamma },c\varepsilon^{2}{\left(X_{\gamma }^{Τ}X_{\gamma }\right)}^{-1}\right)$$

25 $$\pi \left(\varepsilon^{2}\right)=\pi \left(\varepsilon^{2}|\gamma ,X,c\right)\propto \varepsilon^{-2}$$

A diffuse prior on *c* is introduced in eq 26, in which $I_{N^{+}}$ is an indicator function of the set of positive natural numbers, and a uniform prior (eq 27) is imposed on all possible models γ. Because the random variables ε^2^, *c*, and γ are independent of all other parameters of the discrepancy function, their conditional probabilities are equal to their respective marginal probabilities as shown in eqs 25–27.

26 $$\pi \left(c\right)=\pi \left(c|\gamma ,X\right)\propto c^{-1}I_{N^{+}}\left(c\right)$$

27 $$\pi \left(\gamma \right)=\pi \left(\gamma |X,c\right)=2^{-m\times r-1}$$

From the construction of δ in eq 23, the conditional probability of the dynamic discrepancy term at a fixed model structure and other parameters can be reconstructed from eq 24. Specifically, it is a linear transformation of the β_γ_ via the mapping *x*_γ_, which results in the Gaussian distribution in eq 28, and when summed to a zero-mean Gaussian of errors ϵ ∼ *N*(0, ε^2^), results in eq 29.

28 $$X_{\gamma }\beta_{\gamma }|\gamma ,\varepsilon^{2},X,c∼N\left(0,c\varepsilon^{2}X_{\gamma }{\left(X_{\gamma }^{Τ}X_{\gamma }\right)}^{-1}X_{\gamma }^{Τ}\right)$$

29 $$\delta |\gamma ,\varepsilon^{2},X,c∼N\left(0,\varepsilon^{2}\left(I+cX_{\gamma }{\left(X_{\gamma }^{Τ}X_{\gamma }\right)}^{-1}X_{\gamma }^{Τ}\right)\right)$$

The dependency of the conditional probability of δ on the error variance ε^2^ can sum up to a marginal conditional probability by integrating the density function over the domain of ε^2^. The closed-form expression of the probability density functions of π­(δ|γ, ε^2^, *X*, *c*) and *π*(*ε* ^2^|*γ*,*X*,*c*) are respectively given by the Gaussian prior in eq 29 and the Jeffrey’s prior in eq 25. Since the search for the most probable model in the current work is a Markov chain Monte Carlo procedure, a proportion of the model evidence is sufficient for a sampling algorithm. Therefore, the constant factor of the likelihood function in eq 30 is simplified for more efficient computation.

30 $$\begin{array}{ll} & \pi \left(\delta |\gamma ,X,c\right)=\int_{0}^{\infty }\pi \left(\delta |\gamma ,\varepsilon^{2},X,c\right)\pi \left(\varepsilon^{2}|\gamma ,X,c\right)d\varepsilon^{2}\\ & =\int_{0}^{\infty }\frac{{\left(\varepsilon^{2}\right)}^{-n/2-1}}{{\left(2\pi \right)}^{n/2}{\left(c+1\right)}^{-n_{\gamma }+1/2}}\exp\left(-\frac{1}{2\varepsilon^{2}}y^{Τ}{\left(I+cX_{\gamma }{\left(X_{\gamma }^{Τ}X_{\gamma }\right)}^{-1}X_{\gamma }^{Τ}\right)}^{-1}y\right)d\varepsilon^{2}\\ & =\pi^{-n/2}\Gamma \left(\frac{n}{2}\right){\left(c+1\right)}^{-n_{\gamma }+1/2}{\left(y^{Τ}\left(I-\frac{c}{c+1}X_{\gamma }{\left(X_{\gamma }^{Τ}X_{\gamma }\right)}^{-1}X_{\gamma }^{Τ}\right)y\right)}^{-n/2}\\ & \propto {\left(c+1\right)}^{-n_{\gamma }+1/2}{\left(y^{Τ}\left(I-\frac{c}{c+1}X_{\gamma }{\left(X_{\gamma }^{Τ}X_{\gamma }\right)}^{-1}X_{\gamma }^{Τ}\right)y\right)}^{-n/2} \end{array}$$

In the integration over the g-prior to achieve the marginal probability of an output set given a fixed set of basis functions and calibration inputs, the distribution of the prior in eq 27 and the obtained conditional distribution in eq 30 are employed. The indicator function $I_{N^{+}}$ convert the Lebesgue integral in eq 31 to a summation.

31 $$\begin{array}{ll} \pi \left(\delta |\gamma ,X\right) & =\int_{0}^{\infty }\pi \left(\delta |\gamma ,X,c\right)\pi \left(c|\gamma ,X\right)dc=\sum_{c=1}^{\infty }\pi \left(\delta |\gamma ,X,c\right)c^{-1}\\ & \propto \sum_{c=1}^{\infty }c^{-1}{\left(c+1\right)}^{-n_{\gamma }+1/2}{\left(y^{Τ}\left(I-\frac{c}{c+1}X_{\gamma }{\left(X_{\gamma }^{Τ}X_{\gamma }\right)}^{-1}X_{\gamma }^{Τ}\right)y\right)}^{-n/2} \end{array}$$

The probability of the model given the calibration data is obtained from the probability of the generated data given a fixed model by applying Bayes’ theorem. As a result of the uniform prior of the basis structure, the posterior probability of the model is proportional to the likelihood of achieving dynamic discrepancy values, as shown in eq 32.

32 π(γ|δ,X)∝π(δ|γ,X)π(γ|X)∝π(δ|γ,X)

The expansion of the summation in eq 31 is truncated to the first *L*_γ_ terms, and the choice of *L*_γ_ is recommended to be a large number, such as 50. While the proof of convergence is outside the focus of the current work, computational experiments performed confirm the property with scaled data of the BSS-ANOVA Gaussian process for *L*_γ_ greater than or equal to 50. The approximation of the unnormalized model evidence, π̂(γ|δ,X), given in eqs 33 and 34, is employed in the following Gibbs sampling model selection algorithm.

33 π(γ|δ,X)∝π̂(γ|δ,X)

34 $$\mathrm{\pi ̂}\left(\gamma |\delta ,X\right)=\sum_{c=1}^{L_{\gamma }}c^{-1}{\left(c+1\right)}^{-n_{\gamma }+1/2}{\left(y^{Τ}\left(I-\frac{c}{c+1}X_{\gamma }{\left(X_{\gamma }^{Τ}X_{\gamma }\right)}^{-1}X_{\gamma }^{Τ}\right)y\right)}^{-n/2}$$

As the number of calibration data points, *n*, increases, the numerical values of the posterior model probability decrease due to the negative exponent *n*/2. While the Bayesian inference is unaffected since the relative quantities between different probabilities are consistent, floating point error may influence the computation of their values. To avoid this issue, the evaluation of eq 34 is recommended to be used in conjunction with a log transformation to identify the basis and the orders of magnitude.

### 4.2 Gibbs Sampling Procedure

The unnormalized posterior probability of a model, π̂(γ|δ,X), is a combined measure of the data fitness and the model complexity. In fact, a least-squares regression formula will appear if the base of the negative exponent *n*/2 is expanded. The negative exponent $\frac{n_{\gamma }+1}{2}$ penalizes the model with more basis functions if an identical calibration output performance is obtained from two different models. The overall hierarchy of the model evidence strives to achieve different balances between the given calibration data uncertainty and span of the chosen basis functions and between the prior beliefs and the provided data.

If the model evidence is calculated for each dynamic discrepancy model γ, the model with the highest value of posterior probability model is the model that contains a sufficient amount of basis functions with the least chance of being overfitted. However, the number of possible models grows exponentially with the number of basis functions and calibration inputs. Some potential solutions for identifying the best subset of basis functions are using the posterior probability in eq 34 as the objective function for a mixed-integer nonlinear programming problem with the vector of independent variables being γ, or a derivative-free mixed-integer solver such as a genetic algorithm to find the most probable model. By exploiting the probabilistic nature of the model, a Gibbs sampling algorithm is utilized here to iteratively generate a sequence of candidate models, which converge rapidly in distribution to the model evidence.

A detailed description of the steps involved in the Gibbs sampling procedure can be found in Algorithm 1. The inputs of this algorithm are the matrix of calibration inputs provided by eq 22, the calibration outputs δ generated in the previous subsection, the number of samples, *N*_sample_, generated using the Monte Carlo simulation, and the burn-in period length, *N*_burn_. The output of the Gibbs sampling is a sequence of models {*γ*_0_,*γ*_1_,*γ*_2_,...,*γ*_*N*_sample__} that are drawn from the probability π̂(γ|δ,X). For each model, $\gamma_{i}={\left[\gamma_{i,0}\gamma_{i,1}\gamma_{i,2}...\gamma_{i,m\times r+1}\right]}^{Τ}$ , in the sampling sequence, the indicator γ_*i*,*j*_ ∈ 0, 1 denotes the inclusion of the *j*th basis function in the dynamic discrepancy function associated with γ_*i*_.

Using Gibbs sampling, the most likely model is the one that appears with the highest frequency within a sequence of samples generated by the algorithm. To determine the most probable model from the sequence of samples generated, an additional counting algorithm is required, which can be computationally expensive and time-consuming. An alternative solution is computing the probability of inclusion $P\left({\mathrm{\gamma ̂}}_{k}|\delta ,X\right)$ , which is the chance of the *k*th basis function, ${\mathrm{\gamma ̂}}_{k}$ , in the most probable model. Mathematically, the probability of inclusion is defined as shown in eq 35.

35 $$P\left({\mathrm{\gamma ̂}}_{k}|\delta ,X\right)=\frac{\sum_{j=N_{Burn}}^{N_{Sample}}\gamma_{j,k}}{N_{Sample}-N_{Burn}}$$

Some common choices of the threshold for the probability of inclusion are the mean and the median of the probability of inclusion, in which a basis function is considered not important and excluded from the model if its probability of inclusion is below a certain threshold. Furthermore, the probability of inclusion indirectly reflects the quality of the calibration data set as well as the span of the considered basis functions. For instance, if both the calibration data and the considered basis functions are sufficient to recreate the expected performances of the dynamic discrepancy function, there is a clear distinction between the probability of inclusion associated with the basis that should be included and excluded from the most probable model. In contrast, if all probability of inclusions are consistently lower than 50%, the Gibbs sampling sequence is not sufficiently long or the basis functions cannot mimic the dynamic behaviors reflected in the data set.

## 5 Results and Discussions

### 5.1 Case Study: Fischer–Tropsch Synthesis Slurry Bubble Column Reactor

#### 5.1.1 High-Fidelity Dynamic Model Development

The slurry bubble column reactor is assumed to be operated in the churn-turbulent regime to achieve optimal conversion for Fischer–Tropsch synthesis.^42^ In this regime, small bubbles combine into large bubbles and rise up the column in a plug-flow manner at high superficial velocities (in the range of 1–2 m/s). The large bubbles also churn up the slurry phase, allowing the well-mixed assumption to be applicable. The slurry phase contains catalyst particles and small bubbles (1–7 mm in diameter) submerged in paraffin oil, which can be recycled from the product stream of the Fischer–Tropsch synthesis. Vapor–liquid equilibrium is assumed to occur between the liquid paraffin and the gas bubbles, regardless of size. In order to maintain the optimal temperature for the Fischer–Tropsch synthesis reaction, cooling fins are placed internally in the column. The schematic of the developed model is illustrated in Figure 6.

**Figure 6:**
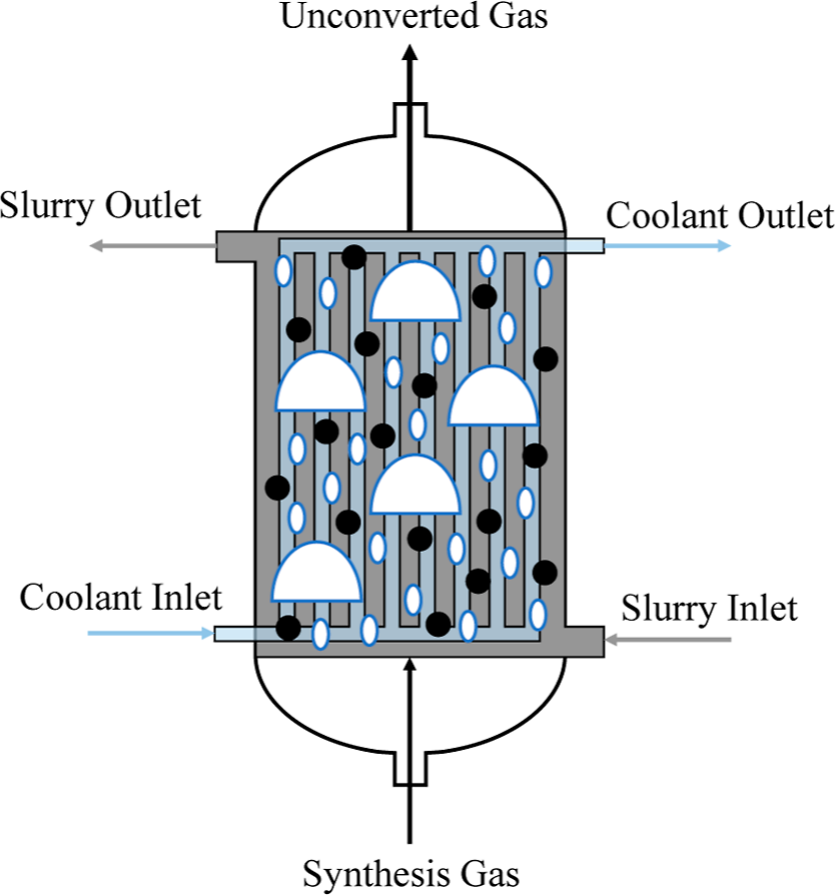
Schematic of the slurry bubble column for Fischer–Tropsch synthesis.

The dynamic model of the slurry bubble column reactor is divided into three phases, and each phase has a separate set of ordinary differential equations to represent its mass balances, energy balances, and thermodynamic properties. The first phase represents the large bubbles rising from the injectors at the bottom of the column. Since these bubbles have a significantly higher rise velocity when compared to the other phases, the large bubble phase is modeled as a plug flow. Furthermore, since the large bubbles also agitate the slurry and the small bubbles, they act as a stirrer and the remaining two phases are assumed to be well-mixed. More specifically, the slurry phase and the small bubble phases are modeled as two continuous stirred tank reactors. A diagram of the modeling approach of the Fischer–Tropsch reactor is shown in Figure 7.

**Figure 7:**
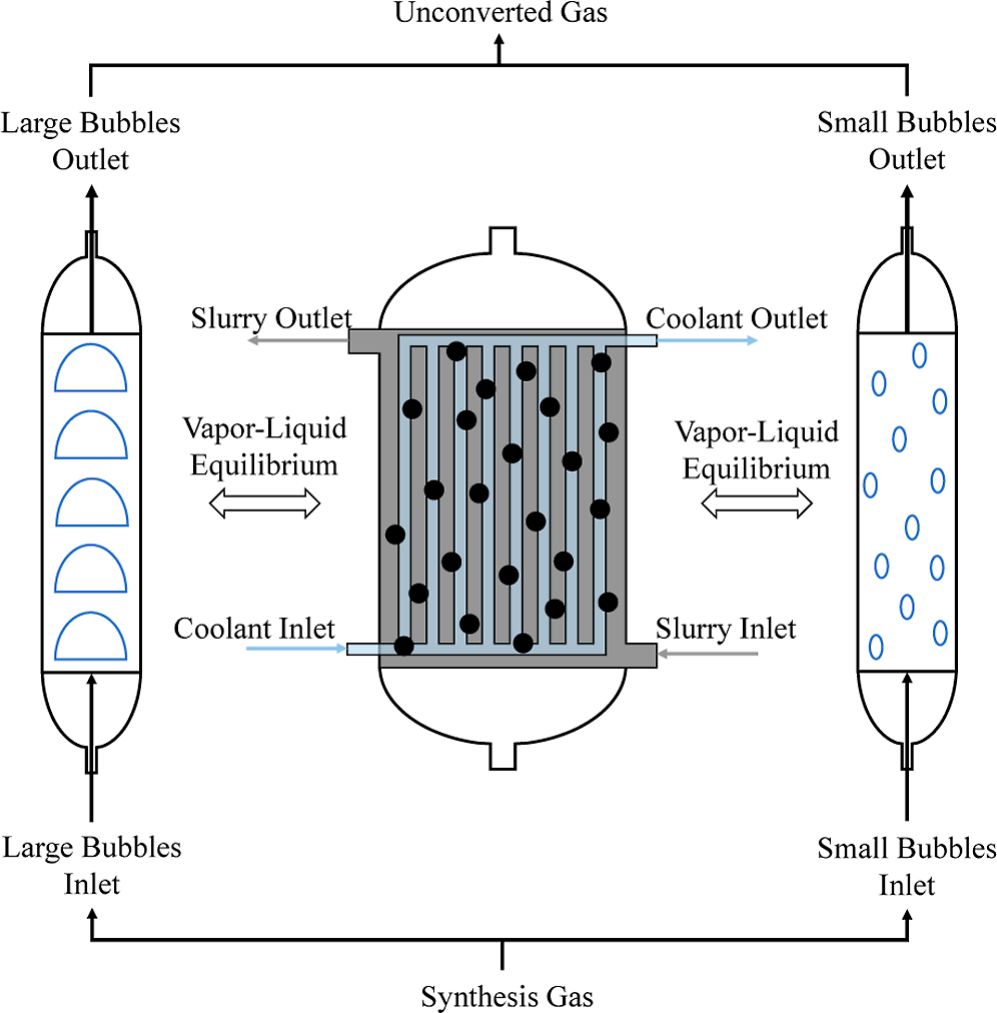
High-fidelity modeling approach for the Fischer–Tropsch synthesis slurry bubble column reactor.

The catalyst is assumed to be a cobalt base with an alumina (Al_2_O_3_) promoter support. This catalyst increases the reaction rate and selectivity of hydrogenation while reducing the water–gas shift reaction to negligible levels. The kinetics of syngas conversion, presented in eqs 36–38, is assumed to be Langmuir–Hinshelwood type, and the mass transfer effect is ignored according to the literature.^43^

36 $$-R_{CO+H_{2}}=\frac{a\times p_{H_{2}}\times p_{CO}}{{\left(1+b\times p_{CO}\right)}^{2}}$$

37 $$a=8.8533\times 10^{3}\times \exp\left[4494.31\left(\frac{1}{493.15}-\frac{1}{T}\right)\right]$$

38 $$b=2.226\times \exp\left[-8236\left(\frac{1}{493.15}-\frac{1}{T}\right)\right]$$

in which $R_{CO+H_{2}}$ is the syngas conversion rate, *a* and *b* are respectively the kinetic parameters, *p*_CO_ and *p*_H_2__ are respectively the partial pressure of carbon monoxide and hydrogen, and *T* is the temperature. The hydrodynamics of the bubbles in the column are obtained through experimental data fitting for the churn-turbulent regime.^44^ The relationships between the gas holdup, ϵ, large bubble holdup, ϵ_b_, small bubble holdup, ϵ_df_, liquid holdup, ϵ_L_, and catalyst holdup, ϵ_s_, can be determined as shown in eqs 39 and 40.

39 $$\epsilon_{L}+\epsilon +\epsilon_{s}=1$$

40 $$\epsilon =\epsilon_{b}+\left(1-\epsilon_{b}\right)\epsilon_{df}$$

The large bubble holdup is affected by the column diameter, *D*_T_, and the superficial gas velocity through the large bubbles, (*U* – *U*_df_), as shown in eq 41, where *U* is the overall superficial gas velocity and *U*_df_ is the superficial velocity through the small bubbles.^44^

41 $$\epsilon_{b}=0.3\times D_{T}^{-0.18}\times {\left(U-U_{df}\right)}^{0.58}$$

the small bubble holdup is a function of gas density, ρ_g_, and the catalyst holdup, as shown in eq 42.^42^

42 $$\epsilon_{df}=0.27\times {\left(\frac{\rho_{g}}{1.2\frac{kg}{m^{3}}}\right)}^{0.48}\times \left(1-2.592\times \epsilon_{s}\right)$$

the increase in catalyst concentration also leads to an increase in small bubble rise velocity, *V*_small_, and an increase in the superficial velocity through the small bubbles, as shown in eqs 43 and 44, respectively.^42^

43 $$V_{small}=\left(0.095\frac{m}{s}\right)\times \left(1+8.421\times \epsilon_{s}\right)$$

44 $$U_{df}=\epsilon_{df}\times V_{small}$$

In the dynamic simulation, performed using Aspen Custom Modeler (ACM),^45^ the column diameter is assumed to be 7.5 m, the overall superficial gas velocity is the manipulated input variable, the catalyst holdup is assumed to be 0.36, and the gas density is calculated using a Peng–Robinson thermodynamic package, with eqs 39–44 solved simultaneously for ϵ, ϵ_df_, ϵ_L_, ϵ_b_, *V*_small_, and*U*_df_.

For each species *c* ∈ {H_2_,CO,H_2_O,C_*n*_H_2*n*_,C_*n*_H_2*n*+2_}, the volumetric mass transfer coefficient between the large bubble and the slurry liquid, ${\left(k_{L}a\right)}_{c,large}$ , is determined as shown in eq 45.

45 $$\left(\frac{{\left(k_{L}a\right)}_{c,large}}{\epsilon_{b}}\right)=\left(0.5s^{-1}\right)\times \sqrt{\frac{D_{c,L}}{2\times 10^{-9}m^{2}s^{-1}}}$$

The volumetric mass transfer coefficients of species *c* between the small bubble and the slurry liquid, ${\left(k_{L}a\right)}_{c,small}$ , is determined by the expression shown in eq 46.

46 $$\left(\frac{{\left(k_{L}a\right)}_{c,small}}{\epsilon_{b}}\right)=\left(1.0s^{-1}\right)\times \sqrt{\frac{D_{c,L}}{2\times 10^{-9}m^{2}s^{-1}}}$$

The diffusivities of the species *c* in the liquid phase, *D*_*c*,L_, are calculated using the Peng–Robinson thermodynamic method assuming the paraffin oil is C_16_H_34_. Let *A* be the cross-sectional area of the bubble column, the dispersion height *H* = 30 m, and the slurry’s super velocity *U*_L_ = 0.01 ms^–1^. ξ_*c*_ denotes the extent of reaction of species *c*, *C*_G,clarge_ indicates the concentration of species *c* in the large bubbles, *C*_G,csmall_ represents the concentration of species *c* in the small bubbles, *C*_L,c_ stands for the concentration of species *c* in the liquid slurry, and *m*_*c*_ is the Henry constant of species *c*. The mass balances at the differential reactor height, d*z*, are represented according to eqs 47–49.

47 $$\begin{array}{l} \frac{d}{dt}\left(\epsilon_{b}\times C_{G,clarge}\right)=-\frac{d}{dz}\left(\left(U-U_{df}\right)C_{G,clarge}\right)\\ -{\left(k_{L}a\right)}_{c,large}\left(\frac{C_{G,clarge}}{m_{c}}-C_{L,c}\right) \end{array}$$

48 $$\begin{array}{l} \frac{d}{dt}\left(H\times \epsilon_{df}\times C_{G,csmall}\right)=U_{df}\times \left(C_{G,csmall}^{i}-C_{G,csmall}\right)\\ -{\left(k_{L}a\right)}_{c,small}\left(\frac{C_{G,csmall}}{m_{c}}-C_{L,c}\right)H \end{array}$$

49 $$\begin{array}{l} \frac{d}{dt}\left(H\times \epsilon_{L}\times C_{L,c}\right)=\int_{0}^{H}{\left(k_{L}a\right)}_{c,large}\left(\frac{C_{G,large}}{m_{c}}-C_{L,c}\right)dz\\ +H{\left(k_{L}a\right)}_{c,small}\left(\frac{C_{G,csmall}}{m_{c}}-C_{L,c}\right)\\ -U_{L}C_{L,c}+H\times \epsilon_{L}\times R_{CO+H2}\times \xi_{c} \end{array}$$

The separation of the three phases is a modeling simplification concept, as the phases are coupled with each other in the actual system. Assuming the heat capacities of the gas phases are negligible when compared to the slurry phase, the energy balance for the reactor is given by eq 50.

50 $$\begin{array}{ll} HA\epsilon_{L}\rho_{s}C_{P,s}\frac{dT}{dt} & ={ṁ}_{H_{2}O}C_{P,H_{2}O}\left[1-\exp\left(-\frac{\alpha_{eff}A_{hx}}{{ṁ}_{H_{2}O}C_{P,H_{2}O}}\right)\right]\\ & +HA\epsilon_{L}R_{CO+H2}\left(\Delta H_{rx}\right)-U_{L}AC_{P,S}\left(T-T_{0}\right) \end{array}$$

The heat exchanging area, *A*_hx_, is assumed to be 6.0 × 10^4^ m^2^, the slurry inlet temperature, *T*_0_, is assumed to be 200 °C. The heat of reaction, Δ*H*_rx_, is calculated by the differences between the specific enthalpy of the reactants and the products. The overall heat transfer coefficient, α_eff_, the slurry density, ρ_s_, and the slurry heat capacity, *C*_P,s_, are calculated according to the literature,^46^ and respectively have the values of 22.23 Wm^–2^ K^–1^, 810 kg m^–3^ and 796 J kg^–1^ K^–1^. The coolant flow rate ${ṁ}_{H_{2}O}$ is a manipulated variable.

The product distribution is assumed to follow the Anderson-Schulz–Flory distribution with the chain growth probability α_rx_. The chain growth probability is used as a controlled variable to manipulate product selectivity. The formula of α_*rx*_ is assumed to be affected by the termination to olefin rate, *T*_olefin_, and the termination to paraffin rate, *T*_paraffin_, as described in eqs 51–53.^47^

51 $$T_{olefin}=6.1686\times P_{H_{2}}^{-0.5}$$

52 $$T_{paraffin}=13.8\times P_{H_{2}}^{-0.47}\times P_{CO}^{-0.43}$$

53 $$\alpha_{rx}=\frac{T_{paraffin}}{1+T_{paraffin}+T_{olefin}}$$

In the following subsection, an NMPC is formulated as a set point tracking controller for the Fischer–Tropsch synthesis bubble column reactor. The manipulated variables are chosen to be the coolant flow rate and the syngas feeding rate. The controlled variables, or the output variables, are the reactor temperature and the chain growth probability of the Fischer–Tropsch reactions. The process disturbance is assumed to be the fluctuation of the slurry concentration in the feed. Full state information is assumed to be available at every time step for a closed-loop control simulation.

#### 5.1.2 Reduced-Order Dynamic Model Development

Since the MPC is model-based, it would be highly dependent on the performance of the process model embedded in its formulation. To test the developed dynamic discrepancy framework, a reduced-order model of the Fischer–Tropsch synthesis bubble column reactor is derived by intentionally removing the large and small bubble phase models from the HFM. Then, the reduced-order model is considered to be only the continuous stirred tank reactor that corresponds to the slurry phase of the full model. The reduced-order model is simplified from the mass balances in eqs 47–49 to the ordinary differential equation shown in eq 54. Since the reactions occur in the slurry phase and on the surface of the catalyst, a submodel of the slurry phase is sufficient to capture the reaction kinetics. However, because the heat and mass transfer are coupled, and considering that all the heat transfer involved is not being properly accounted for due to the missing bubble phases in the ROM, it is expected that both the heat transfer and mass transfer in the reduced-order model behave differently from the HFM.

Additionally, instead of considering a full set of species *c* ∈ {H_2_,CO,H_2_O,C_*n*_H_2*n*_,C_*n*_H_2*n*+2_} with the length of the hydrocarbons between 1 and 30, the ROM considers a pseudocomponent (i.e., a lumped hydrocarbon mixture) to replace the paraffins and olefins presented in the HFM model. The product specification is recreated using the Anderson-Schulz–Flory distribution and the concentration of the pseudocomponent.

54 $$\frac{d}{dt}\left(H\times \epsilon_{L}\times C_{L,c}\right)=-U_{L}C_{L,c}+H\times \epsilon_{L}\times R_{CO+H2}\times \xi_{c}$$

The reactor temperature and chain growth probability profiles in open-loop for both the high-fidelity model and reduced-order model are shown in Figure 9. These profiles were obtained assuming a common fixed manipulated input sequence in terms of the cooling water flow rate and gas superficial velocity for both models considering a time horizon of 200 min, as shown in Figure 8. The results show a completely different dynamic behavior for both the temperature and chain growth probability, which consequently leads to different steady states. The observed results highlight the insufficiency of the reduced-order model in representing all the undergoing heat and mass transfer of the Fischer–Tropsch synthesis and, thus, the need for a discrepancy term to account for the mismatch between the reduced-order and high-fidelity models for incorporation into the NMPC.

**Figure 8:**
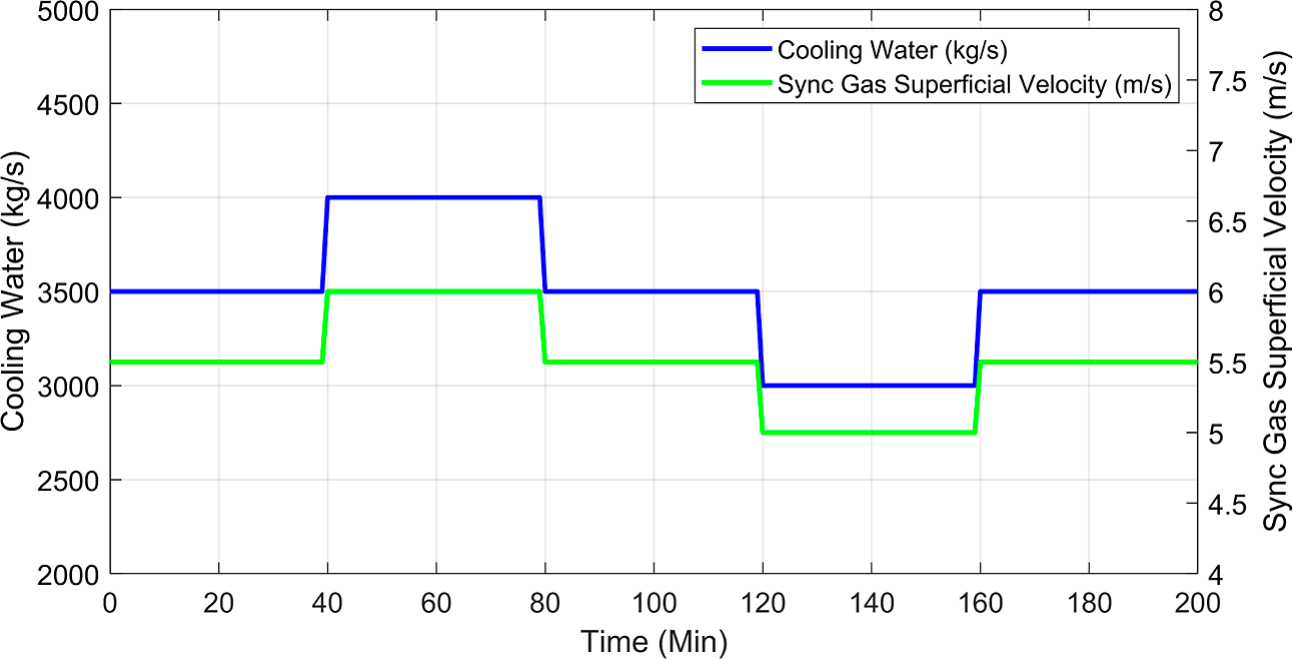
Manipulated input sequence for the water flow rate and superficial gas velocity in open-loop simulation.

**Figure 9:**
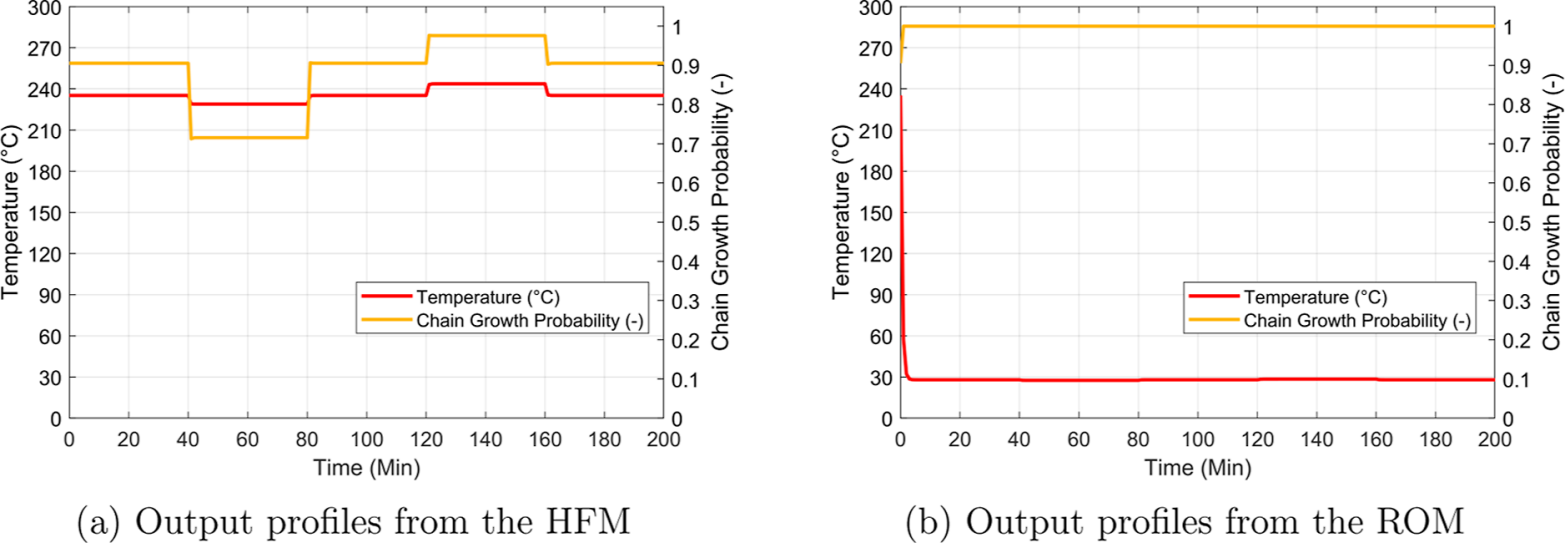
Temperature and chain growth probability profiles from both the HFM and ROM models.

#### 5.1.3 DD-ROM Calibration and Closed-Loop Simulation Results

The reduced-order model considered for the Fischer–Tropsch synthesis bubble column reactor is augmented by the discrepancy functions. Thus, a dynamic discrepancy reduced-order model (DD-ROM) is obtained, as shown in eq 55. This DD-ROM is expected to properly capture both the reaction kinetics and the temperature regulation. This happens because the cooling fins only establish contact with the slurry during the computation of the heat exchange area, so the slurry phase is expected to behave similarly to the HFM model.

55 $$\begin{array}{ll} \frac{d}{dt}\left(H\times \epsilon_{L}\times C_{L,c}\right) & =\delta \left(C_{L,c},U_{L},{ṁ}_{H_{2}O}\right)\\ & -U_{L}C_{L,c}+H\times \epsilon_{L}\times R_{CO+H2}\times \xi_{c} \end{array}$$

In the HFM of the Fischer–Tropsch synthesis process, the plug-flow characteristic of the large bubble phase is modeled by a discretization along the height of the slurry bubble column using the method of lines. Therefore, each species in the large bubble phase is represented by a vector of state variables that correspond to a specific location in the reactor, and the number of differential equations increases proportionally with the number of considered species. Because the DD-ROM only accounts for the slurry phase, which is assumed to be well-mixed due to the rise of the large bubbles, each species in this phase only requires one differential equation to represent these dynamics.

The set of basis functions for the dynamic discrepancy in eq 54 are the basis of the BSS-ANOVA Gaussian process obtained in Subsection [Sec sec3.4]. For each calibration input, a set of BSS-ANOVA basis functions up to order 4 are generated, and cross interactions between basis functions of different calibration inputs are also considered. In total, there are 210 basis functions, and the number of possible models is 2^211^ ≈ 3.3 × 10^63^, which is computationally intractable to estimate. Thus, Algorithm 1 is applied with the Markov chain length of 100,000 and a burn-in length of 5000. The most probable model is the model that only contains the basis functions with probabilities of inclusion higher than 50%. The validation data set confirms the fitness of the obtained DD-ROM, and a fraction of the validation result is shown in Figure 10. An important property observed from the validation data set is that the calibration errors do not accumulate over time because the formulation of the dynamic discrepancy function satisfies the proposed criteria in Section 3.2.

**Figure 10:**
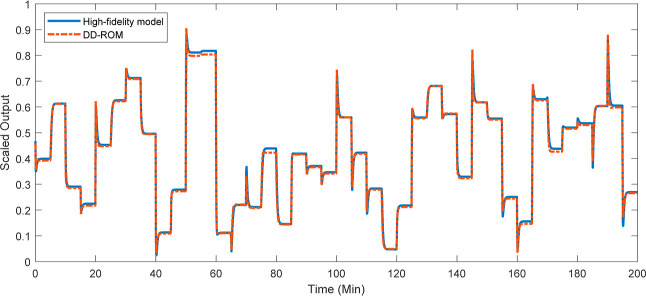
Validation of the DD-ROM for the Fischer–Tropsch synthesis reactor.

The performance and computational efficiency of using both the HFM and the DD-ROM as equality constraints for the NMPC optimization are compared. First, the model performances are evaluated considering the controller objectives of set point tracking and disturbance rejection. The prediction horizon of the NMPC is selected to be 20 min, optimized every 6 s. Additionally, to test the capabilities of the closed-loop process, a new output set point vector is generated every 5 min to demonstrate the NMPC can bring the system to any achievable outputs in a finite time.

The input profiles obtained during the MPC optimization using both the HFM and the DD-ROM as equality constraints in the NMPC are shown in Figure 11. From these profiles, one can observe that the solutions of the NMPC with HFM and the NMPC with DD-ROM are similar, with a slightly spikier superficial gas velocity profile for the DD-ROM, which does not affect the controller’s ability to reach the desired targets nor compromise the applicability of the proposed framework. The slightly spikier superficial gas velocity observed in the DD-ROM stems from using MPC tuning parameters originally calibrated for the HFM, which results in more aggressive input actions. Although this behavior could be reduced by retuning the controller for the DD-ROM, the parameters were intentionally kept identical to ensure a fair comparison.

**Figure 11:**
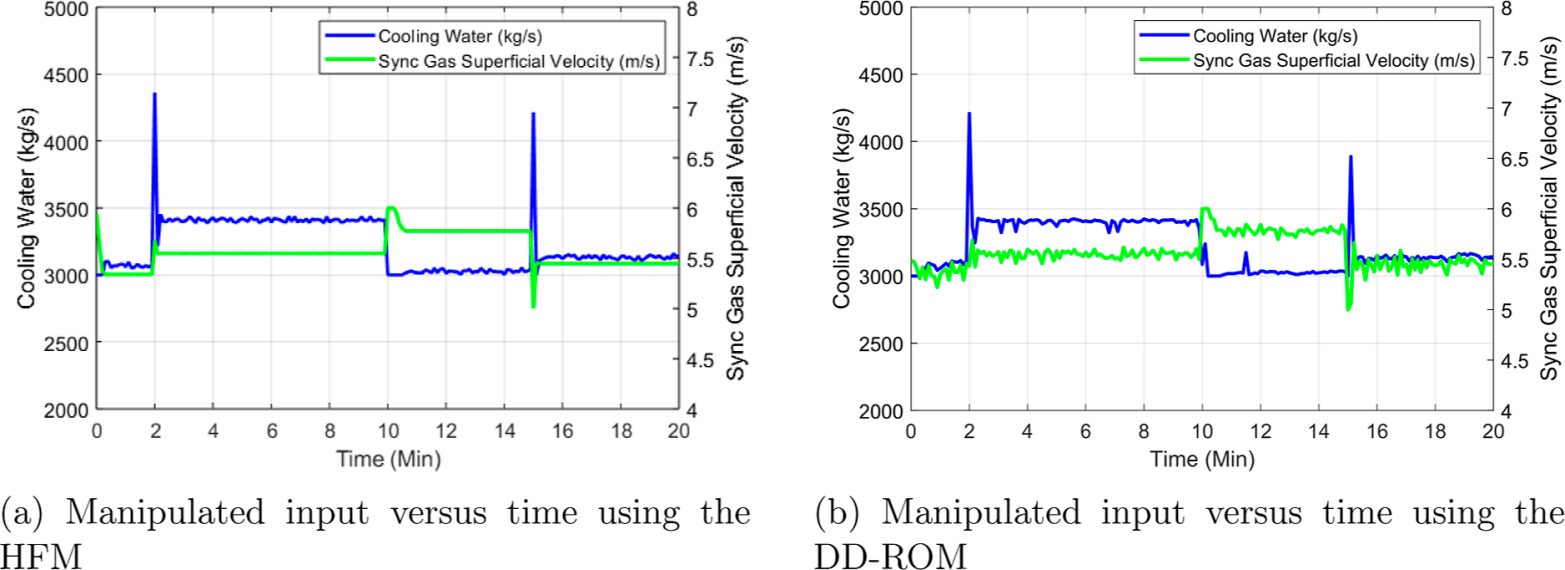
Optimized input sequence obtained during the MPC optimization using both the HFM and DD-ROM models within the NMPC.

Regarding the system output, the temperature and chain growth probability profiles for both the HFM and DD-ROM are shown in Figure 12. From these profiles, one can see that the controller can bring the plant models to the desired outputs successfully for every case using both the DD-ROM and HFM models, with an *R* ^2^ of 0.99 and 0.97 for each output, respectively, when comparing the output profiles in Figure 12.

**Figure 12:**
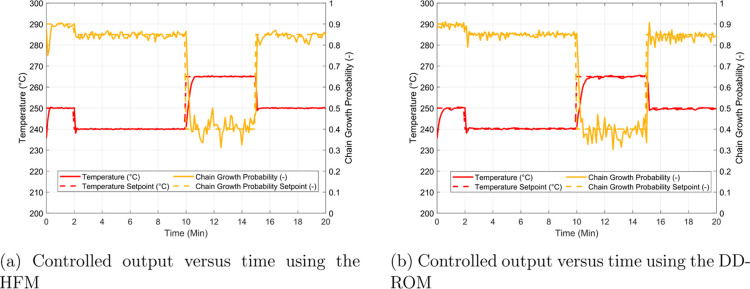
Temperature and chain growth probability profiles using both the HFM and DD-ROM models within the NMPC.

In terms of computational efficiency during the NMPC optimization, it is possible to see in Table 1 that the DD-ROM outperformed the HFM model as expected. All case studies were performed on a PC with the following configuration: 32 GB RAM, Intel Xeon E5–1620 v3 processor. While the total CPU time for the whole optimization performed running the NMPC using the DD-ROM was 929 sec, the same optimization task using the HFM required a total time of 1314 sec. Regarding the average CPU time per optimization step, the DD-ROM required 4.65 sec, while the HFM model required 6.57 sec. These results show that using the DD-ROM as the process model reduces the average CPU time per optimization by approximately 2 sec, corresponding to a 41.3% improvement in computational efficiency relative to the HFM. The modest gain in this case is explained by the convergence behavior of the MPC closed-loop problem in Aspen Custom Modeler. Once the system reaches a set point, both the HFM and the DD-ROM converge rapidly, which limits the relative speedup. Despite this, in other case studies, especially those involving slower system dynamics, the computational gains of the DD-ROM can be substantially more significant.

**Table 1:** Comparing the Computational Efficiency of the HFM vs. DD-ROM Used as MPC Models During Optimization

model	total CPU time for optimization (s)	average CPU time per optimization (s)	improvement in computational efficiency (%)
HFM	1314	6.57	-
DD-ROM	929	4.65	41.3%

Moreover, based on the reported average CPU time per optimization, one can see that the optimal solutions of the online nonlinear programming problem of the NMPC with the DD-ROM are successfully solved within the 6 *s* limit indicated by the average CPU time per optimization of the HFM, demonstrating the potential feasibility of the NMPC DD-ROM to be applied in an actual process. All these results highlight the advantages of the dynamic discrepancy framework proposed in this work.

## 6 Conclusions

In this work, a novel framework for dynamic discrepancy reduced-order modeling formulation was introduced for advanced control applications. The framework consisted of three independent steps. The first step was formulating the dynamic discrepancy terms in the rate of change equations, and three essential criteria were proposed as heuristics for implementations. The second step was generating the calibration data, which was proposed using a moving horizon estimator. The last step was a Bayesian inference to simultaneously estimate the hyperparameters and select a model structure according to Occam’s razor principle. The dynamic discrepancy function introduced significantly improves the reduced-order model obtained by compensating for the differences in the rates of change instead of the time-varying outputs of the ROM. During the formulation, the study presented a series of heuristics as criteria for constructing a DD-ROM that was suitable for advanced control applications. Moreover, a moving horizon data collection procedure was proposed that enhances the flexibility of placing the discrepancy function in the ROM, while simplifying the calibration process to a linear Bayesian model selection. The approach considered the construction of the posterior of the model given the calibration data and employed a Gibbs sampling procedure to search for the most probable model when the number of possible models was extensive due to a high number of potential basis functions. The framework was successfully applied to the closed-loop control of the Fischer–Tropsch slurry bubble column reactor, and the results obtained through the application of DD-ROM showed that it fits for practical implementation.

The proposed methodology for developing the DD-ROM for NMPC exhibits high adaptability and can be utilized fully or partially in numerous systems. This work thus lays a foundation for the future development of a gray-box modeling approach with dynamic discrepancy. Specifically, the incorporation of the dynamic discrepancy functions within the ROM is independent of the calibration of the function. As a result, other data-driven models, such as a neural network, can be implemented instead of the BSS-ANOVA Gaussian process for the DD-ROM. Alternatively, the proposed calibration with Gibbs sampling can be improved with a model prior that accounts for the significance of each basis function. For future process systems engineering research, a more in-depth guideline for the placement of the discrepancy functions in the gray-box model could be developed based on the physical phenomena, and an online calibration approach could be established in combination with state estimation methods.
